# End-of-life and palliative care of patients on maintenance hemodialysis treatment: a focus group study

**DOI:** 10.1186/s12904-019-0481-y

**Published:** 2019-10-30

**Authors:** Lena Axelsson, Eva Benzein, Jenny Lindberg, Carina Persson

**Affiliations:** 1 Department of Nursing Science, Sophiahemmet University, Box 5605, 114 86 Stockholm, Sweden; 2 Centre for Collaborative Palliative Care, Faculty of Health and Life Sciences, Linnaeus University, Kalmar, Sweden; 3 Department of Clinical Sciences, Unit of Medical Ethics, Faculty of Medicine, Lund University, Lund, Sweden; 4 Department of Nephrology, Skåne University Hospital, Malmö, Sweden

**Keywords:** End-of-life care, Palliative care, End-stage kidney disease, Hemodialysis, Physicians, Nurses, Focus group interviews

## Abstract

**Background:**

Despite complex illness trajectories and a high symptom burden, palliative care has been sub-optimal for patients with end-stage kidney disease and hemodialysis treatment who have a high rate of hospitalization and intensive care towards end of life. There is a growing awareness that further development of palliative care is required to meet the needs of these patients and their family members. In this process, it is important to explore healthcare professionals’ views on provision of care. The aim of this study was therefore to describe nurses’ and physicians’ perspectives on end-of-life and palliative care of patients treated with maintenance hemodialysis.

**Methods:**

Four focus group interviews were conducted with renal nurses (17) and physicians (5) in Sweden. Qualitative content analysis was used to analyze data.

**Results:**

Participants were committed to giving the best possible care to their patients, but there were challenges and barriers to providing quality palliative care in nephrology settings. Professionals described palliative care as end-of-life care associated with hemodialysis withdrawal or palliative dialysis, but also identified care needs and possibilities that are in line with an earlier integrated palliative approach. This was perceived as complex from an organizational point of view. Participants identified challenges related to coordination of care and different perspectives on care responsibilities that impacted symptom management and patients’ quality of life. Communication issues relating to the provision of palliative care were revealed where the hemodialysis setting was regarded as an impediment, and personal and professional experiences, beliefs and knowledge were considered of major importance.

**Conclusions:**

Nurses and physicians identified a need for the improvement of both late and earlier palliative care approaches. The results highlighted a requirement for and possibilities of training, counselling and support of health care professionals in the dialysis context. Further, multi-professional palliative care collaborations should be developed to improve the coordination and organization of end-of-life and palliative care of patients and their family members. A climate allowing conversations about advance care planning throughout the illness trajectory may facilitate the gradual integration of palliative care alongside life-prolonging treatment for improved support of patients and families.

## Background

The need for palliative care to enhance wellbeing and quality of life for persons with a life-threatening disease and their families has been emphasized [1]. It is also recognized that a palliative approach involving the adaptation of palliative care expertise and knowledge [2] needs to be integrated into different contexts in order to reach patients with chronic life-limiting diseases, e.g. nephrology care [3, 4]. The need for integrated palliative care that meets the care needs of persons with chronic non-malignant diseases is also emphasized by the Swedish National Board of Health and Welfare [5]. In Sweden there is a policy that general palliative care, performed by all health care professions, should be provided regardless of the care contexts when the patients have palliative care needs. Moreover, professionals from specialized palliative care should be contacted and/or consulted when needs are complex [5]. How this is organized and fulfilled for patients with advanced renal disease varies with different renal departments.

End-stage kidney disease (ESKD) is life-limiting with high mortality rates [6] and patients have complex illness trajectories with several comorbidities and a high symptom burden [7, 8]. Moreover, their situation is vulnerable due to suffering related to treatment hardships, feelings of being a burden and ambivalence about life and death [9]. The end-of-life trajectories may also involve dialysis withdrawal followed by an average survival of a few days [10]. Through the whole illness trajectory, family members face increasing responsibilities involving decision dilemmas and they need support to be able to cope [11].

The situation for these patients is therefore difficult, yet palliative care has been suboptimal for patients with ESKD receiving dialysis treatment and they have a high rate of hospitalization [12] and intensive care needs towards end of life [13]. Even if death is expected, patients have unmet palliative care needs [14]. Furthermore, family members have rated the end-of-life care of these patients as significantly worse than that of e.g. patients with cancer or dementia [15]. Dialysis professionals have also reported lack of quality care, with unmet needs such as end-of-life care discussions and bereavement support [16].

The barriers to timely start of palliative care may be the uncertain prognosis of chronic diseases, lack of practical knowledge and complex decision-making [16, 17]. In addition, the patient’s trajectory towards death in a hemodialysis context may be intricate for the healthcare professionals who have often developed a close relationship with patients during dialysis treatment which can occur 3–4 times/week over several years. At the same time as facing issues such as patients’ suffering, withdrawal decisions and death, physicians and nurses may be preoccupied with advanced medical technology [18] with a focus on standard care metrics, which can jeopardize a holistic view of the patient, patient priorities and end-of-life decisions [19].

There is a growing awareness that development of palliative care is required to meet the needs of patients with ESKD and their family members [3, 4, 20]. Further research is needed to integrate palliative care into the context of hemodialysis treatment. In this task, healthcare professionals’ views of the significance of palliative care in this context is essential for a successful adaptation. Therefore, the aim of this study was to describe nurses’ and physicians’ perspectives on end-of-life and palliative care of patients treated with maintenance hemodialysis.

## Methods

### Design

The study had a qualitative descriptive design using focus group interviews [21].

### Participants and procedure

Participants were recruited from two hospitals in Sweden using purposive sampling [22]. Inclusion criteria were being a physician, registered nurse or enrolled nurse working in a hemodialysis unit and/or other renal ward and with at least 1 year of experience of caring for patients on maintenance hemodialysis. Eligible participants were identified and approached by the study contact nurse or the head nurse in collaboration with one of the researchers (LA). In total, 22 healthcare professionals participated: 5 physicians, 15 registered nurses and 2 enrolled nurses. (Hereafter, registered nurses and enrolled nurses are called “nurses”.) All participants worked in nephrology settings in county hospitals; either in hemodialysis units/centers or renal nursing wards. Some worked in both the dialysis unit and the nursing ward. Participants’ ages varied from 27 to 61 (mean 45) years. Their professional experience ranged from 4 to 37 years (mean 19). All physicians were specialists in nephrology or had renal training and all nurses had renal training in the nephrology setting. Their experience of caring for patients on hemodialysis treatment varied between 1 and 31 years (mean 12).

### Data collection

Data were collected from January to February in 2016. Four focus groups [21] were conducted with a mix of physicians and nurses and with five to six participants in each group. All participated on one occasion. The interviews were led by a moderator and an assistant moderator (CP, LA). One moderator had earlier clinical experience as a dialysis nurse, one was an experienced researcher in palliative care. The interviews took place in a quiet private room close to the dialysis units. Using open, circular and clarifying questions, the participants were encouraged to reflect and discuss their perspectives of palliative care and the care of severely ill patients treated with hemodialysis and approaching end of life (e.g. “Please tell us your experiences of caring for severely ill patients on maintenance hemodialysis approaching end of life”; “what are your thoughts on palliative care of patients on dialysis”; can you tell us more about that?) They were encouraged to talk freely and to interact with and respond to each other during the interviews with questions as “what do you think when you hear what your colleague says”. Question areas in the interview guide were generally spontaneously raised by participants. The first focus group interview was considered a pilot test of interview questions and we found that questions generated rich data hence the interview was included in the study. The audio-taped interviews (total 7.5 h) were transcribed verbatim.

### Data analysis

Qualitative content analysis with an inductive approach was used [23, 24]. The moderators (CP, LA) discussed the content of the interview directly after each interview and made field notes. When all interviews had been performed, they were listened to by the moderators and the interview texts were then read several times. “Meaning units” (i.e. parts of sentences, sentences or paragraphs each containing a meaning) corresponding to the aim of the study were identified, condensed, coded (LA, CP) and thereafter discussed, revised and sorted into sub-categories and categories (LA, CP). The identified patterns [23] were discussed within the research group until agreement was reached.

## Results

The participants described complexities and challenges in the care of severely ill patients approaching end of life. They expressed perceptions of palliative care in a renal context, varying from end-of-life care in the very last days of life to the care of someone whose diagnosis will eventually lead to death. Among the different perceptions of palliative care, there was a mutual understanding that patient-defined quality of life should decide the direction of care. During the interviews, the participants showed commitment to providing quality end-of-life care and articulated a need for earlier onset of palliative care components corresponding to a palliative approach, without explicitly talking about this approach. They described impediments to this task and expressed a need for more knowledge and support. The analysis resulted in the overarching theme *Perceiving palliative care as end -of-life care whilst also identifying patients’ needs for earlier integration of a palliative care approach*.

Findings are presented as three categories and ten sub-categories which are presented in Table 1.

**Table 1:** Categories and sub-categories

Understanding of palliative care in the hemodialysis context	Palliative care means dialysis withdrawal and end-of-life care Palliative dialysis means focusing on quality of life rather than medical parameters
Challenges of palliative and end-of-life care	Relations and changing functions End-of-life communication Support of family members Symptom management Organization of care of patients with palliative care needs
End-of-life care involves a need for follow-up conversations	Post-bereavement meetings with family members Information to co-patients Follow-up conversations for the staff

### Understanding of palliative care in the hemodialysis context

#### Palliative care means dialysis withdrawal and end-of-life care

Palliative care of patients treated with hemodialysis was predominantly expressed as end-of-life care during the last weeks or days of life and was usually performed on the nephrology in-patient ward. Palliative care was also expressed as being equivalent to care given after a decision to withdraw dialysis. It was stressed that this situation is unique for this context since death will usually occur within a few days. Palliative care was also recognized as the initiation of individual prescriptions needed for end-of-life symptom management. However, one physician reflected that maintenance dialysis is always in a sense palliative, but that using the expression palliative care only for the care of patients close to death facilitated a mutual understanding of the term in the nephrology context. The expression “palliative patient” was used and explained as follows:

*A person whose illness is not reversible and will inevitably lead to death. And, in a way, I see dialysis as a form of palliative care, because they will of course die of kidney failure or something else. But for me, it is more that - death is now imminent. (Focus group interview (FGI) 1)*

#### Palliative dialysis means focusing on quality of life rather than medical parameters.

Palliative care was also related to “palliative dialysis”, that is when the seriously ill patient is still on maintenance dialysis treatment, but with treatment goals being aimed at quality of life. A physician said: *“then there is no reason to treat aggressively, the patient must be allowed to rule/…/ but the medical parameters are no longer in focus”. (FGI 4).* Palliative dialysis involved a reduced dialysis regimen regarding frequency and/or duration of treatment sessions, a phase that often preceded dialysis withdrawal. Although the goal of quality of life was clarified, treatment decisions could be complex, e.g. palliative dialysis that is initiated to enable more time at home could lead to an increase of symptoms and therefore lessen quality of life in other ways. Hence, it was further stressed that palliative dialysis is a “double-edged sword” (FGI 3) that may even prolong suffering. Physicians emphasized that palliative dialysis should not mean abandoning the patient in the subsequent complex decision-making, since expertise in nephrology is still needed.

### Challenges of palliative and end-of-life care

#### Relationships and changing functions

Participants emphasized their capability in relation to palliative and end-of-life care due to the often long and close relationships with the patients. Physicians also described a challenge in their change of professional functioning associated with the transition from nephrology to end-of-life and palliative care. They reported that it could be difficult “to let things be”. There were also emotional challenges when accompanying a patient over a long illness trajectory until the end. However, physicians considered that showing emotions risked giving the impression of a lack of professionalism. Beliefs that physicians may avoid the dying patient due to uncertainty regarding their role in end-of-life situations were expressed. A physician said:

*During end of life we do not visit the patients as much in the nursing ward. We are afraid that we cannot offer any more help. What can we do in a situation with a dying patient? (FGI 2).*

But a need for more time for physicians for bedside end-of-life care was also stressed.

The nurses did not clearly identify any change in their professional activities when adopting palliative care, but it emerged that it was often the nurses who initiated discussions in the care team about dialysis withdrawal and palliative care, since they identified the patients’ suffering and diminished quality of life. After decisions on dialysis withdrawal, the nursing goal was emphasized as making the last days of life as valuable as possible for the patient and family.

#### End-of-life communication

Communication about end-of-life and existential issues was regarded as challenging and difficult to initiate, although this was facilitated by life experience. A physician experienced that when he initiated conversations with severely ill patients, the patient had usually already had inner feelings about his/her severe situation, which supported the need for the conversation. End-of-life communication as supportive communication was described as being primarily a matter of attentive listening and being open to the patients’ thoughts. One physician pointed out that merely listening may be difficult though since their profession and time demands active dialogue. A nurse said:

*I think that everybody, or anyway I find talking about death difficult. It is a loaded subject, you are afraid of saying the wrong thing or misunderstanding someone, it’s difficult. Of course at some point you’ve heard someone say that they are afraid of dying and …We know of course that it’s important to listen, but it is so very hard. I think we’re not used to it. (FGI 1).*

Participants thought that, in the hemodialysis unit where the focus is on life-sustaining treatment, they may try to keep death at a distance, although death is always close. A nurse said:

*Our relationship with death is complex. At the same time as we say that death is part of life,*” *you shouldn’t be afraid” – we say that if you do this or that then you will die. We are conflicted in the way we view death. (FGI 3).*

The participants identified a need for earlier conversations with patients regarding their understanding of and wishes regarding end of life. Hence, participants expressed a need for more pro-active communication. A physician and a nurse reflected:

*Physician (P): you can ask them “when the time comes to stop dialysis, how do you want things to be* (the others agree by saying yes, yes!).

*Nurse (N): Yes, “how do you want the last phase of your life to be?” Yes…*

*/…/*

*P: But how many doctors and nurses ask the patient this?*

*I don’t think that we have talked to any patient on dialysis about… how they want the last phase of life to be.*

*N: No...(FGI 2).*

Still, there was no routine for these conversations, which instead depended on the individual nurse or physician’s readiness for such conversations. The dialysis setting was also regarded as an impediment for end-of-life conversations. Some participants believed that the patients did not want to take the time for conversations in a private room. A nurse who also worked in home care (with peritoneal dialysis) pointed out that, during home visits in peritoneal dialysis care, patients and their family members often talk about death in a natural way when talking about the future. Hence, differences in the settings’ potential for end-of-life communication were clarified.

Knowing the opinions and wishes of the patient concerning end of life and treatment early on was regarded as being helpful, since e.g. later decisions about dialysis withdrawal may be taken without the patient being able to participate. However, participants acknowledged that end-of-life wishes may change over time and communication should therefore be an on-going process. The participants discussed the importance of knowing how the patients perceived their quality of life and suggested that regular patient-reported measurements together with active listening may facilitate communication with patients about their life-situation and goals of care towards end of life. The significance of early end-of-life planning to enable dying at home more often was emphasized by a physician who gained insights when once visiting a patient at home.

*The difference between when he was on the ward in the hospital environment and when we visited him at home, and the clear authority that he had in his own home, where we were guests and he could talk, and he was in control, and it was his things, and he could offer us coffee, that was dignity, in my view. It left a huge impression of how it could be when at its best. Because he ... he was on home ground. (FGI 1).*

It emerged that most decisions about place of death were taken late when the patient may not be able to express an opinion or when it was believed that a dignified death would be facilitated if the patient stayed in the hospital. A physician reflected that although the patient and/or family should always decide the place of death, the physicians may well influence their decision in favor of the nephrology ward, since care at home may be very demanding.

#### Support of family members

Participants acknowledged the burdened situation of family members, particularly after dialysis withdrawal. Giving them support in their exposed situation at end-of-life was stressed as an important component of palliative care. A physician emphasized that communicating the meaning of palliative care with family members is important:

*I usually say that palliative care does not mean inferior care. It means that we should help as much as we can and we can do a lot. And this improves the whole situation. (FGI 2).*

Participants also reflected that, despite long treatment duration and that illness and dialysis treatment also affects close family members, they seldom get to know the family. A nurse emphasized that knowing the dying patient through the continuity of dialysis treatments brought a feeling of trust and comfort to family members when meeting them later on the nephrology ward. Family members feeling prepared and acknowledged was also underlined as a factor in a dignified death. The family members are usually invited to an end-of-life discussion when questions of dialysis withdrawal arise, but participants identified that they could and should be invited earlier in the patient’s illness trajectory.

#### Symptom management

Symptom management was regarded as a prerequisite for a dignified death and the nurses experienced that, when decisions were taken about palliative care, it was easier to obtain the necessary prescriptions. This helped them to feel confident in delivering quality end-of-life care. A lack of and need for regular symptom assessment using validated symptom scales was suggested and discussed during interviews. A physician said: “these scales can show if we have made any misjudgments, we can find out about things that we weren’t aware of.” *(FGI 4).* The enrolled nurses’ observations of subtle signs in the dying patients were also underlined as being important for avoiding patients’ suffering, and nursing assessment with documentation was stressed by physicians as being important for appropriate symptom management. However, the nurses emphasized that the most difficult areas of symptom management, such as managing severe pain, may exist long before death, i.e. due to the often complex symptom burden in severely ill patients there was a need for improved symptom management in general, not only at the end of life.

#### Organization of care of patients with palliative care needs

The challenges of the organization and co-ordination of care were identified and discussed since participants experienced that the care management of severely ill co-morbid patients may “fall between” care units and specialists (sometimes located in different towns). The humanity in the organization was questioned when nurses experienced a gap in patient care but it was stated (by nephrologists) that in the hemodialysis unit they “should only deal with dialysis” and other matters were not their commission. This was problematized as the issue of responsibility being complex. A physician reflected:

*There are problems when there are gaps in the structure and organization of healthcare, no-one can hold the reins. It is reasonable that the dialysis unit holds the reins because dialysis is given there 3 times per week, but I think the main problem is somewhere else in the organization. (FGI 4).*

The importance of palliative care as organized care was stressed by a nurse:

*Palliative care means taking a step back and listening to the patients and their family members, showing that we are there for them. They should know that there is a plan and that they have someone to turn to. They should not have to struggle with different healthcare contacts. (FGI 4).*

End-of-life and palliative care were mainly considered to be tasks for renal physicians and renal nurses, and it emerged that patients were seldom enrolled in specialized palliative care. Participants emphasized that palliative care requires engagement and time. Nevertheless, at the in-patient renal ward, the quality of care also depended on the general workload situation.

### End-of-life care involves a need for follow-up conversations

#### Post-bereavement meetings with family members

The needs of family members do not end with the death of the patient but routines for post-bereavement meetings with family members varied between units. Some aimed to offer all family members a meeting. Others offered a follow-up when they considered it important to elucidate the situation around the patient’s death. A physician reflected that, if more time was spent with the patient and family before death, then bereavement support might not be needed as much. However, a nurse stressed that family members may have thoughts or questions that the professionals are not aware of so a meeting should always be offered. A physician and nurse reflected:

*P: I had a lovely meeting with NN’s husband, he was really pleased with everything, apart from that fact that her personal belongings were in a bag that he thought looked like a bin bag, and he took that really hard. Yes, that is something that …that we just shouldn’t do.*

*N: I also think that we can do things … through thoughtlessness, that have an enormous impact on the family members’ experiences. (FGI 1).*

#### Information to co-patients

A concern in the dialysis context was whether, or how, to inform co-patients about a patient’s death. The close relationships between patients in this context complicated these decisions and the participants had varying perceptions about this multifaceted issue. Participants also had different views on the legal aspects. It appeared that a person’s death is usually followed by silence in the dialysis unit. Some stressed that such information may cause anguish in co-patients and others believed that not talking about it might cause more anguish and that patients are anyway confronted with death. A physician reflected that these situations might be an opportunity to initiate conversations about end-of-life wishes with co-patients. Participants also discussed points both for and against making a statement about a patient’s death in some way, for example lighting a candle to respect the person whose life had ended.

A physician reflected:

*It is quite an interesting discussion on the dialysis ward, patients go there for years and meet their fellow patients and the staff who are fairly consistent throughout this time, of course there are other aspects to this. And sometimes, professionalism may be a bit rigid, it’s a balance. And how do we deal with this? We can’t be too unfeeling either and we definitely can’t sweep it under the carpet without saying anything. (FGI 3).*

#### Follow-up conversations for the staff

Both nurses and physicians reflected on their own needs to gather and talk about the deceased person and the end-of-life care given. This was raised as both a professional and a personal need after a long care relationship.

*You can try to stay professional and see them just as patients, but in reality it’s not like that. You become very close to some patients over a long period of time. And it feels important to sit down and and talk about …your feelings now that this person is gone. (FGI 1).*

The nurses had opportunities to support each other and receive professional guidance but the physicians may feel alone in the exposed and complex situation of end-of-life care and its necessary decisions. The participants reflected:

*P: It is never anything structured and we rarely have the time or opportunity to sit down on the ward and talk about a patient that has died /…/ like now, to gather and get a sense of: How did you feel, or what did you think, and should we have done anything differently?*

*N: What can we take to the next case?*

*N: This is something we don’t do. Light a candle and sit down and talk. (FGI 1).*

On the other hand, a need to prioritize time with patients and family members before death rather than spending it on a meeting with colleagues after the death was also stressed.

## Discussion

The results of this Swedish focus group study, of the perspectives of renal healthcare professionals on end-of-life and palliative care of patients treated with maintenance hemodialysis, show that physicians and nurses were clearly committed to giving the best possible care for their patients, but there are challenges and barriers in nephrology settings to providing quality palliative care. This is both at an early stage and at the end of life. Findings revealed tension between different comprehensions of a palliative care approach for patients on hemodialysis that could hamper early patient-centred interventions to manage symptoms and achieve quality of life. There was a sense of dichotomy between life-prolonging interventions and a palliative approach, also linked to dialysis withdrawal, which is not in line with integrated palliative care. The connection of palliative care and renal care, also named renal supportive care is a more extensive approach which includes patients struggling with dialysis and patients on conservatively managed care i.e. care that may last for years [3, 4].. Renal supportive care is endorsed as part of nephrology [4]. Perceptions of palliative care may be an issue of knowledge and up-to-date palliative care education but still, present findings revealed that also with knowledge of palliative care this term may be used for end-of-life care and dialysis withdrawal as this is the traditional use of the term in the dialysis context. This suggests a challenge for the organization and attitude in the nephrology context. Considering the high mortality and morbidity rates together with the high and complex symptom burden in patients with ESKD, an earlier integrated palliative care approach is likewise called for by the Dialysis Advisory Group of the American Society of Nephrology [19]. In their definition, they recommend a palliative approach for the last year of the patients’ life. Furthermore, the Advisory Group defines a palliative approach in this context as “a transition from a conventional disease-oriented focus on dialysis as rehabilitative treatment to an approach prioritizing comfort and alignment with patient preferences and goals of care to improve quality of life and reduce symptom burden”.

In the present findings, it was clear that professionals recognized this transition, but it was also perceived as a complex task related to various treatment strategies (e.g. ordinary dialysis regime, palliative dialysis, dialysis withdrawal) with different effects on symptoms and on the possibilities for professionals to manage these in order to not reduce patients’ quality of life. The dilemma between quality of life vs. various treatment strategies as well as quality indicators has been acknowledged and more studies on different palliative dialysis approaches and their impact on quality of life have been recommended to make dialysis a more person-centered treatment not merely guided by biochemical results [19].

The transition from a predominantly life-prolonging treatment intention to a more palliative approach was also perceived as complex from an organizational point of view. Findings emphasized the need for re-organization and collaboration between different caregivers for improved palliative care of these patients, both earlier and at the end of life. These areas for improvement with regard to the care of patients with palliative care needs are also highlighted internationally and nationally [3, 19, 25] suggesting the need for a mutual understanding between different caregivers of the process of providing good palliative care. This involves advance care planning including all caregivers and access to palliative care specialist/counselling teams [25]. There are models of care that identify ways to handle challenges to integrate palliative care for patients with advanced kidney disease. For example, in St George hospital in Australia the renal supportive care service includes a palliative care physician and a palliative care nurse employed within the department of nephrology [26]. Scherer at al [27, 28] describe a program of integrated early and late palliative care with an ambulatory kidney palliative care team in New York including a physician trained in both nephrology and palliative care and a palliative care psychologist. These and other examples show that there is a worldwide progress in the understanding and practice of integrated palliative care.

Present findings also highlight communication issues related to the provision of quality palliative care where the setting was regarded as an impediment, but where personal and professional experiences and beliefs were also considered of major importance. Linked to the definition of a palliative care approach which emphasizes alignment with patient preferences and goals [19], communication is required to advance individualized care. The present findings point to a dilemma; although the participants identified a need for conversations with patients about end-of-life issues, these were regarded as challenging and difficult to initiate. Also previous studies have shown that conversations about end of life are often postponed due to professionals’ sense of unpreparedness for serious conversations, fear of causing the patient distress and uncertainty regarding the appropriate timing for the patient [29, 30]. Present findings also showed that conversations depended on the individual professional’s readiness. This further adds to the communication dilemma, since some physicians expressed a complex challenge regarding communication, emotions and their professional responsibilities. This type of challenge has also been recognized in previous studies indicating that nephrologists do not find themselves sufficiently well prepared for end-of-life care and communication [17, 31, 32]. A survey of members of the European Renal Association-European Dialysis and Transplant Association (ERA-EDTA) found that palliative care had not been part of nephrologists’ specialist curricula or their continuous medical education [33], which might explain some of the challenges physicians expressed in the present study.

Communication challenges seemed, according to the present findings, to result in postponed conversations which frequently resulted in decisions being taken at a stage when the patient could no longer participate in the decision-making process. This does not correspond to a palliative care approach and is not in line with patient self-determination. From the perspective of patients with ESKD, conversations about end-of-life preferences have been found to be appreciated [34, 35] and studies in different contexts have shown that patients rely on healthcare professionals to initiate this type of conversation [30, 34, 36]. The decisional burden on family members might also be reduced by initiating conversations while the patient is still capable of participating.

Participants in the present study reflected that family members should also be involved in conversations earlier in the illness trajectory. Likewise, bereaved family members have expressed wishes for earlier invitations to discuss the present and future illness situation regarding deterioration while on hemodialysis [11, 37] and also suggested that healthcare professionals have a responsibility to include aspects other than medical ones in conversations e.g. emotional and social-practical issues [37]. This seems to be of great importance to target the holistic goals and general quality indicators of palliative care, such as quality of life for patients and families [1], but healthcare professionals might need more education and support to be able to initiate and perform such conversations according to present findings.

It came forth in present findings that communication on end-of-life occurred more often in home-visits (performed in peritoneal dialysis care) which suggests the significance of the setting. It is also found that patients may prefer home visits for serious illness conversations and end-of-life planning [38]. Family caregivers have similarly described the importance of a safe and unhurried environment for these conversations [37]. In the present study it came forth that visiting the hemodialysis patient at home may also be appreciated by nephrologists, but in Sweden these visits are rare and not part of hemodialysis care practice.

Findings show that routines vary concerning bereavement support. Yet, to offer post-bereavement meetings at dialysis centers has come forth as important to family members [11]. There are in Sweden regional renal care guidelines including bereavement support but still this support of family members is often lacking [14].

Findings also illuminate the complex issue of communicating a co-patient’s death, or not. This issue resulting in “silence” in the hemodialysis unit after a patient’s death has been described by co-patients as being left alone with questions [39].

### Methodological considerations

Strengths and limitations are discussed according to criteria for trustworthiness [23]. Credibility was strengthened by using purposive sampling strategy. This resulted in participants with varying experiences which contributed to rich data and transferability within similar contexts, although the data material is limited to two hospitals in Sweden. Hence, findings may be of relevance to health care professionals in renal and palliative care in other countries, as reflected by international research. Dependability was addressed by discussions among coauthors [23] (all females) where the authors’ different preunderstandings of the context (two of the researchers, one nurse and one physician, have clinical experience of renal context) and of palliative care (clinical experience and/or research) contributed to critical examination throughout the analysis process. The voices of the participants are made visible through the quotations in the results section, which will also be helpful when judging the relevance of interpretations and, hence, readers will be able to assess the confirmability of this study.

Participants’ interpersonal and inter-professional interactions during mixed focus group interviews contributed to an understanding of the topic in this context as multifaceted and engaging and is considered a strength of the study. During interviews, the climate was permissive, and the participants challenged and stimulated each other’s reflections and input. After the four interviews we considered that data was sufficient for analysis of results as data was recurrent and rich. The moderator in this study has vast experience of performing group discussions, which buffered the possible limitations of using mixed focus groups (cf. [40]).

Since this study involves only professionals in nephrology care, the results need to be confirmed by further studies where the hemodialysis patient’s perspectives are evaluated. We have referred to some previous studies on the subject but we recognize the need for further anchoring in the patient experience when clarifying the goals for future nephrological palliative care.

## Conclusions

This study contributes to the growing literature on the need for development and integration of person -centered palliative care principles and pro-active end-of-life care of patients in hemodialysis care. Nurses and physicians in the hemodialysis context identified needs for improvement of both end-of-life care and an earlier adoption of a palliative care approach. This requires a common terminology for palliative care to be able to adopt palliative care even at an early stage of maintenance hemodialysis. Professionals also face challenges regarding palliative care of these patients in a context including both acute and end-of-life care. The study highlights the needs and possibilities for training, counselling and support of nurses and physicians in the dialysis context. Multi-professional palliative care collaborations should also be developed to improve the coordination and organization of end-of-life and palliative care of the patients and their family members. Findings emphasize the importance of creating a climate in the hemodialysis setting that makes conversations about advance care planning and end-of-life more natural throughout the illness trajectory in order to meet the emergent need of patients to discuss these issues. This may facilitate a gradually integrated palliative care alongside life-prolonging treatment which may also contribute to fulfilling patient preferences regarding the end of life. Home visits to hemodialysis patients may be one way to improve conversations in order to gain a more holistic view of the patient and family, and their specific needs when introducing palliative and end-of-life care. This is an area for further research. Other recommendations for further research are bereavement support and information to co-patients in the dialysis unit after a patient’s death.

## Data Availability

Not available due to ethical regulations.

## Abbreviations

ESKD: End-stage kidney disease
FGI: Focus group interview
N: Nurse
P: Physician
